# Testing a Multidimensional Acculturation Model on Latinos who Completed Substance Abuse Treatment

**DOI:** 10.21767/2471-853X.100023

**Published:** 2016-05-24

**Authors:** Roberto Lopez-Tamayo, J Alvarez, LA Jason

**Affiliations:** Center for Community Research 990 W. Fullerton Ave, Suite 3100, USA

**Keywords:** Multidimensional acculturation, Substance abuse treatment, Latinos, Substance abuse recovery, Structural equation modeling, Structure regression analysis

## Abstract

Disparities in substance abuse treatment (SAT) utilization and completion suggest that traditional substance abuse models may fall short of addressing the complex needs of Latinos, increasing the odds of relapsing. The need for substance use models that integrate multiple acculturation domains in relation to substance abuse is warranted. The goal of the present study is twofold: a) test a multidimensional acculturation model on Latinos who completed SAT; and b) examine the association between the proposed multidimensional acculturation and days consuming alcohol and illicit drugs in the past six months. A total of 131 participants (Mage=36.15, SD ± 10.5, 86.3% males, 48.1% non-U.S. born with a mean length of stay of 19 years in the U.S. (SD ± 13.71) were interviewed upon completion of SAT. Results from the SEM indicated adequate model fit to the population under study, supporting the use of a multidimensional acculturation approach for this population. Subsequent results from the structured regression analysis suggest that multidimensional acculturation is negatively associated with days using alcohol in the past 6 months. The implications of these findings are discussed.

## Introduction

Substance abuse completion rates among Latinos are affected by systemic and cultural factors that increase the odds for relapse [1]. The sparse literature on substance abuse treatment (SAT) utilization among Latinos indicates that Latinos encounter more barriers to services [2, 3], reported that their needs were not met in treatment [4], and are more likely to drop out from SAT than their European American counterparts [5]. These results suggest that traditional substance abuse models may fall short of addressing the complex needs of Latinos who complete SAT [6–8].

Among the factors implicated in substance abuse, acculturation has been associated with increased rates of alcohol and drug use substance abuse on Latinos [9–13]. Substance abuse literature has documented existing disparities in substance abuse within Latinos based on level of acculturation and other factors that impact acculturation including years of residence in the U.S. and generational status [5, 14, 15]. Given that substance use is a behavior informed by the individual’s values and social practices (i.e., rules, norms, customs), a close examination of multiple dimensions implicated in the acculturation process is warranted.

Acculturation theorists propose a multidimensional framework to examine different domains in relation to the outcome of interest [16–19]. Based on a multidimensional approach, acculturation is defined as “the confluence among heritage-cultural and receiving-cultural practices, values, and identities” [20]. The cultural clash signified by the differences in values and social norms that prevent Latinos from adapting successfully to the U.S. mainstream culture, within a specific environment, may lead to different behavioral consequences including substance use relapse [21]. However, most studies measuring acculturation on Latinos used data from nonclinical samples [12] and employed unidimensional markers including country of origin [22], length of stay in the U.S. [15] and language [23, 24]. The examination of acculturation using unidimensional measures may mask acculturation aspects that may be relevant for the recovery process.

Of importance is the study of higher-order constructs in substance use recovery. Studies on social control [25] and social learning [26] indicate that substance use behavior can be partially explained as a response to identification with permissive environments that debilitate the individual’s self-efficacy [27]. Studies using community samples of Latinos indicate that U.S. born Latinos have higher rates of substance use than Latino immigrants [15, 28–30]. However, the more years Latino immigrants spent in the U.S., the more they resemble their U.S. born counterparts in terms of substance abuse behavior [9, 31, 32]. Taken together, the inclusion of cultural orientation in substance use models is needed to further our understanding on specific acculturation aspects that may be linked with substance abuse. More important, research in this area is critical to inform and develop sustainable and effective substance abuse aftercare for Latinos [21].

Although the Substance Abuse and Mental Health Services Administration calls for the incorporation of cultural aspects at all different stages of substance use prevention and treatment (i.e., screening, assessment, placement, aftercare and recovery services, program development, and research), intake assessments and discharge planning may overlook individual needs that are important for substance use recovery process, particularly for Latinos [5]. Mounting evidence supports the use of substance abuse models to identify acculturation domains associated with substance use. By the same token, research in this area would provide service providers and researchers the information to develop and implement culturally-tailored treatments and services for Latino substance users [1, 14]. Thus, the purpose of the present study is twofold: a) test a multidimensional acculturation framework [20] that integrates endorsement of social practices (i.e., behavioral acculturation), perceptions and attitudes toward the home and the U.S. mainstream culture (i.e., attitudinal acculturation) and generational status (i.e., immigrant, U.S. born from immigrant parents) on Latinos who completed SAT, and b) examine the association between a comprehensive acculturation measure and substance use in the past 180 days. The proposed multidimensional acculturation framework is hypothesized to render more exploratory power than using simplistic unidimensional acculturation models [20, 33]. Having a better understanding of the association between multiple acculturation dimensions and substance use would help design and implement effective substance use relapse interventions for Latinos working on their recovery [21].

## Methods

### Participants

Participants for this study were part of a larger NIH-funded study that examined community-based recovery homes for Latinos in recovery from substance abuse [34]. A total of 131 Latinos were recruited from multiple substance abuse treatment programs and health facilities from a large metropolitan area in the Midwest. The criteria for participation were 1) being of Latino background, and 2) either having completed substance abuse treatment or living in a controlled environment. There were 131 Latino participants (M_age_=36.15; *SD* ± 10.5), 113 males (86.3%) and 18 females (13.7%). Nearly half of the participants immigrated from Puerto Rico, Mexico and other Central American countries (48.1%), with a mean length of stay of 19.2 years (*SD* ± 13.71) in the United States. The majority of the participants had alcohol and substance abuse treatment previously (n=124), while for seven participants it was their first time in treatment. For socio demographic characteristics **(see **Table 1).

### Setting and procedures

Recruitment of participants took place from fall 2009 to spring 2012. A group of bilingual/bicultural Oxford House alumni and research assistants was formed to facilitate outreach, recruitment and assessment of Latino participants. Research assistants utilized internet search engines (i.e., Google, Yahoo) and statewide databases of health services and mental health providers to generate a list of substance treatment programs, hospitals, and community-based agencies servicing Latinos. The outreach strategy consisted of contacting these sites via phone and email to introduce the study. A team of Oxford House alumni, two of them Latinos, worked to establish ties with staff and potential participants at various treatment centers. Recruiters provided information on community-based recovery home options, described the nature of the study to potential participants, and facilitated the interview process. All participants were given an explanation about the nature, purpose and goals of the study before signing consent forms. Participants were interviewed in their language of preference (i.e., English or Spanish). Interviews took place at treatment facilities, a private location within an Oxford House, or at the DePaul Center for Community Research. After completing the interview, participants received $30 as a compensation for their participation.

## Measures

### Demographics

A 24-item demographic questionnaire was used to collect participants’ age, gender, country of origin, and parent’s country of origin.

### Acculturation

A generational status composite was computed using the participant’s country of origin and the participant’s parents’ country of origin. The following categorization was employed to assess participant’s exposure to the U.S. mainstream culture: Latino immigrant (1) U.S. born Latino with both immigrant parents (2) U.S. born with an immigrant parent and a parent born in the U.S. (3) U.S. born Latino with both parents born in the U.S. (4) Higher generational status indicates more exposure to the U.S. mainstream culture.

The Bidimensional Acculturation Scale for Hispanics [35] is a 24-item, 4-point Likert-type (1=low or not well to 4=high or very well) self-report measure of English and Spanish use as a proxy for acculturation. Three subscales measure language use, linguistic proficiency, and use of electronic media subscales in both Spanish and English. An item sample of the language subscale includes “how often do you speak English?” The Hispanic and Non-Hispanic domain scores are derived from the total scale, where scores higher than 2.5 suggest biculturalism. Good to high internal consistency (α=0.81–0.97) and high correlation with other behavioral measures of acculturation, such as generation in the U.S. and proportion of life spent in the U.S. are reported [35]. For the present study the Hispanic and the Non-Hispanic subscales were used to assess for behavioral acculturation. Higher scores indicate more endorsement of either Latino-oriented behaviors or U.S. mainstream behaviors.

The Psychological Acculturation Scale [36] is a 10-item, 9-point Likert-type scale (1=only with Latinos to 9=only with Anglos) self-report measure that assesses sense of attachment to and belonging within the U.S. and Hispanic/Latino cultures. An item sample includes “with what group of people do you feel you share most of our beliefs and values?” A mean total score is derived from the scale, where a score of 5 indicates bicultural orientation. Both the English and Spanish versions of the PAS have good internal consistency (α=0.90 and 0.83) and correlate with language and cultural preferences, along with percentage of life spent in the U.S. and measures of cultural values [37]. The PAS has been used with a sample of Mexican Americans, Central Americans, and South Americans and found to be correlated with both the proportion of life spent in the U.S. and measures of cultural values [37]. The mean total score was used for the present study to assess participants’ perception of both their home (ethnic) culture and the host (U.S. mainstream) culture.

### Substance abuse

The Form-90 [38] was utilized to reconstruct daily alcohol and substance use consumption within a 180-day time span. The three primary outcome measures of the Form-90 that we will use are drinks per drinking days, percentage of days abstinent, and total number of days of illicit drug use. Days in which participants reported using alcohol or illicit drugs in the last 180 days were coded with a “1”; and days on which participants did not use alcohol or illicit substances were coded with a “0.” The Form-90 was translated into Spanish using translation and back-translation procedures by a team that included a professional translator, a psychologist, and a psychology graduate student. The Form-90 had been used in several studies with Hispanic/ Latino samples to produce valid data [39–41]. A count index of days using alcohol and illicit substances in the last 180 days will be used for the present study. Higher scores will indicate greater alcohol and illicit drug use severity.

## Results

Preliminary analyses, using pairwise deletion to address the issue of missing data, were conducted to determine descriptive statistics. The final sample used for the model analysis was 131 participants (*n*=63 immigrant, *n*=68 U.S. born), with a mean age of 36.15 years. Means, standard deviations and correlations for all study variables are presented in Table 2. Bivariate correlations indicate that, psychological acculturation is positively correlated with affiliation to the U.S. culture, higher generational status, and with being younger and being male. Affiliation to the U.S. culture is negatively correlated with affiliation to Latino culture, lower generational status, fewer days consuming alcohol in the past 6 months, and being younger and being male. Conversely, affiliation to Latino culture is positively correlated with alcohol consumption in the last 180 days, being older and being a male **(see **Table 2).

**Figure 1:**
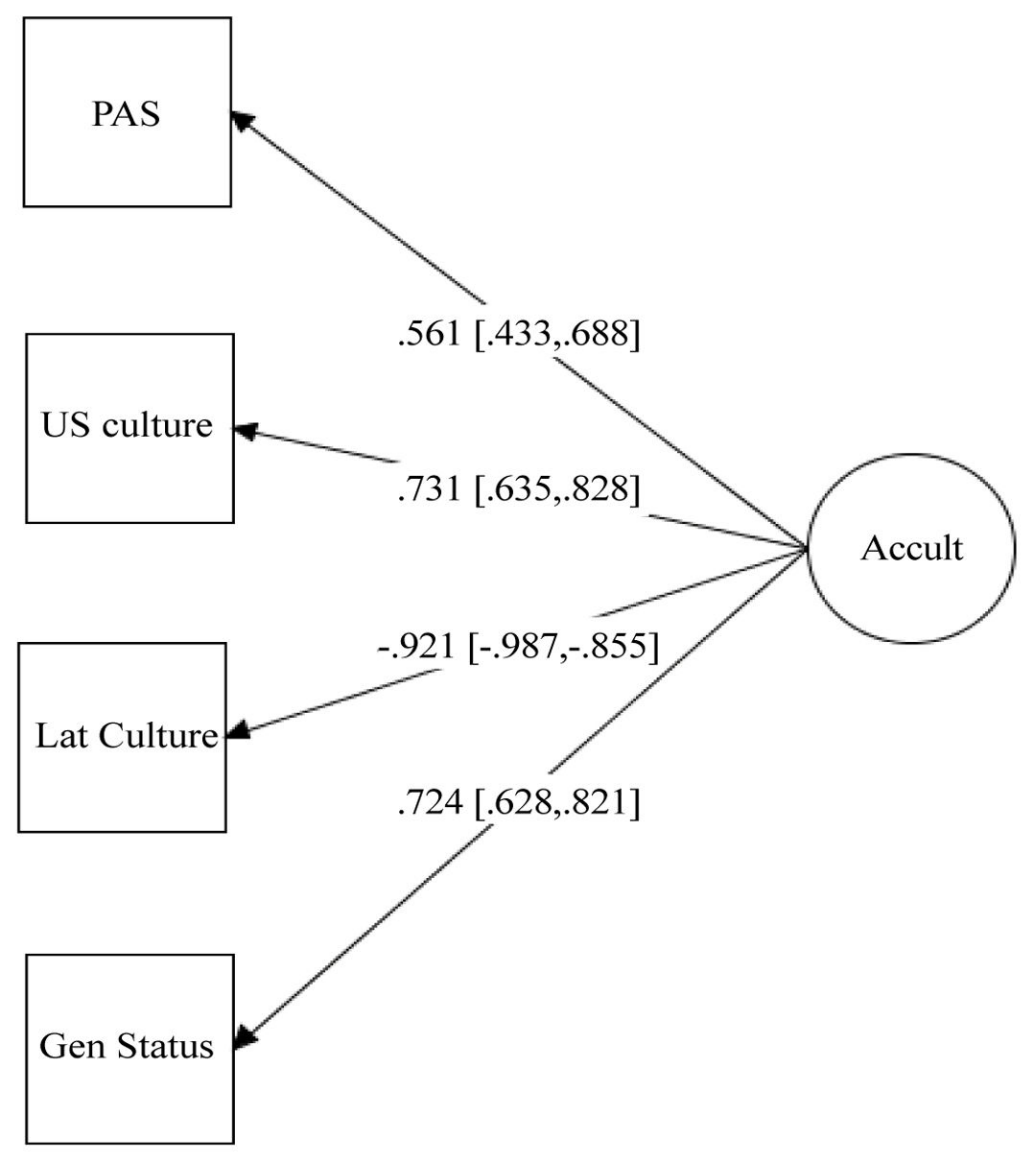
Standardized Estimate for the Structural Equation Model.

The hypothesized multidimensional model was tested using the Mplus computer software, version 7.2 [42] Structural equation modeling (SEM) with maximum likelihood estimation procedures was employed to create a composite of acculturation derived from the means of the Hispanic and non-Hispanic subscales from the BAS (behavioral acculturation), the mean of the PAS (attitudinal acculturation) and generational status. Several different fit indices, including chi-square, the comparative fit index (CFI), the Tucker-Lewis Index (TLI) and the root-mean-square error of approximation (RMSEA) were examined to assess the fit of each model. The CFI is an index that compares the specified model with a model, with the assumption that all variables are uncorrelated. The CFI and TLI range from 0 to 1 and values greater than .95 are considered indicative of adequate fit. The chi-square is a statistical test of “badness of fit,” which is influenced by the model’s degrees of freedom. The RMSEA is an index that is not influenced by model complexity and a value of .08 or less will be consistent with acceptable model fit The SRMR is the standardized average of the covariance residuals and values of 0.10 or lower are indicative of acceptable fit [43] (Figure 1).

Results from the SEM model with paths freely estimated displayed adequate model fit indices (*X*^2^=9.23, *df*=5, *p*=0.10, CFI=0.98, TLI=0.96, RMSEA=0.07, RMSEA 90% C.I.=0.00–0.15, SRMR=0.03). This model accounted for more variance in the behavioral domain, particularly in the Latino orientation. Then, a two-step modeling was employed to test for structural component [44]. A model with generational status constrained to zero was tested and yielded poor model fit indices (*X*^2^=85.74, *df*=6, *p*=0.00, CFI=0.64, TLI=0.40, RMSEA=0.31, RMSEA 90% C.I.=0.25–0.37, SRMR=0.22). The chi-square from the trimmed model was compared to the chi-square from a model with paths constrained to equal. The two models were significantly different (Δχ^2^=77.06, Δ*df*=1, *p*=<0.001), suggesting that the hypothesized model with paths freely estimated is more parsimonious.

**Figure 2:**
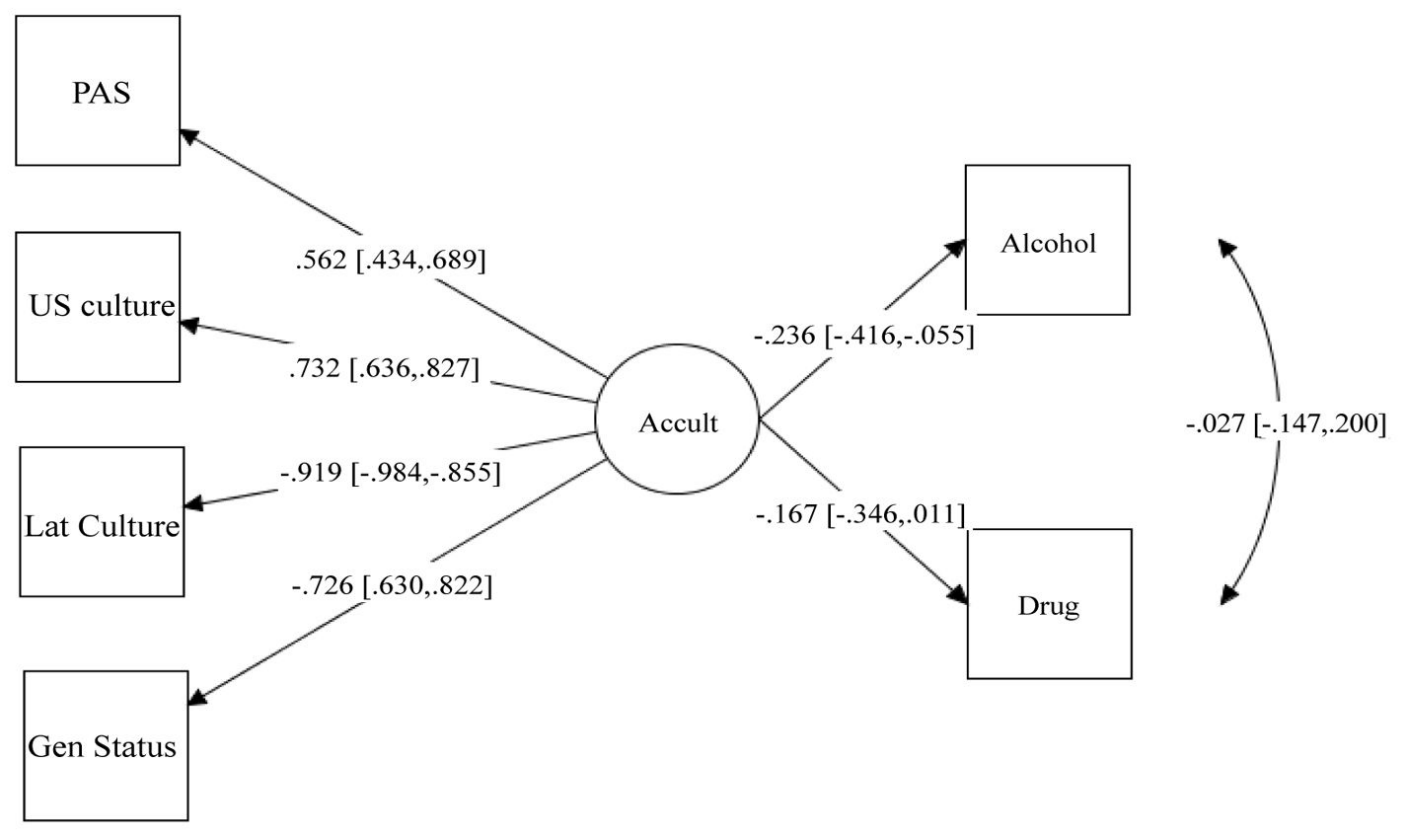
Standardized Estimates for the Structure Regression Model.

Subsequently, structural regression (SR) analysis with maximum likelihood estimation procedures was used to regress alcohol and substance use in the past six months on a latent multidimensional acculturation. Structural regression combines confirmatory factor analysis and path analysis models to test hypothesis about directional effects. Results from the regression model showed good model fit for all fit indices (*X*^2^=25.78, *df*=17, *p*=0.09, CFI=0.97, TLI=0.95, RMSEA=0.06, RMSEA 90% C.I.=0.00–0.10, SRMR=0.07). Then, after controlling for age and gender, acculturation was significantly negatively associated with alcohol use in the past six months (b=−0.24, *p=*<0.01), whereas acculturation was marginally associated with drug use in the past six months (b=−0.17, *p=0*.05). In other words, for each 1 standard deviation (SD) increase in multiple dimensions of acculturation, there is a −0.24 SD decrease in days using alcohol in the past six months. The magnitude of the path between acculturation and alcohol use indicate a small effect. To test the measurement model, a nested model with the path from acculturation to drug use was constrained to zero was conducted. The nested model yielded poor fit indices (*X*^2^=35.44, *df*=18, *p*=0.01, CFI=0.93, TLI=0.89, RMSEA=0.09, RMSEA 90% C.I.=0.04–0.13, SRMR=0.08). The chi-square from the constrained model was compared to the chi-square from a model with paths freely estimated. The two models were significantly different (Δχ^2^=9.66, Δ*df*=1, *p*=<0.001), suggesting that the model with paths freely estimated yielded a better fit to the data (Figure 2).

## Discussion

The purpose of the study was to test a multidimensional acculturation model by examining the measurement model of the structural equation model, and the association between multidimensional acculturation and alcohol and substance use in the past six months.

Results from the structural equation model indicated adequate model fit to the population under study, supporting the use of behavioral acculturation (i.e., affiliation to the U.S.culture, affiliation to Latino culture), attitudinal acculturation (i.e., psychological acculturation), and generational status (i.e., degree of exposure to the U.S. mainstream culture) to generate a comprehensive acculturation measure. Results from the current study advances the substance abuse literature on Latinos by testing a multidimensional acculturation model on a clinical sample of Latinos who completed substance abuse treatment. Of significance is that the proposed framework accounted for more variance in the Latino-related social practices, followed by participants’ affiliation to the U.S. mainstream culture and generational status. Although attitudinal acculturation explains less variance in the model than the other domains, the SEM analysis supports its contribution in the assessment of such complex construct. Overall, the proposed multidimensional model (i.e., behavioral and attitudinal acculturation, generational status) rendered a valid acculturation composite for this population.

The finding from the structure regression analysis indicating that Latinos who completed SAT with more Latino culture orientation, more U.S. culture orientation, and higher generational status (i.e., immigrant, U.S. born with immigrant parents, U.S. born with a U.S. born parent) reported fewer days using alcohol in the past 180 days. These results expand on existing literature on alcohol use among community samples of Latinos [13, 15]. Higher affiliation to the Latino culture serves as a protective factor against alcohol use. However, other acculturation domains seemed to contribute to this association. It is plausible that the average length of Latino immigrants living in the U.S., which is 19 years, may explain the higher affiliation to the U.S. mainstream culture. This finding is also consistent with the immigrant paradox [13, 31], which posits the longer Latino immigrants live in the U.S., the more they resemble their U.S. born counterparts in relation to substance abuse rates [15].

Conversely, our finding indicating that acculturation was marginally negatively associated with days using drugs during the past 6 months deserves consideration. A plausible explanation for the non-significant association is that most participants reported using alcohol and, to a lesser degree, only drugs, or alcohol and another drug. Studies that employed larger samples of individuals who accessed or completed SAT have more statistical power to explain the hypothesized associations [45].

There were several limitations, including a small sample that did not allow for testing model invariance for each measure (i.e., BAS, PAS) or disaggregation of the sample into subgroups (i.e., immigrant vs. U.S. born). By the same token, due to sample size restrictions, the current study was unable to use other covariates in the model (i.e., history of incarceration, employment pattern, social support) that potentially influence substance abuse. The cross-sectional nature of the present study does not allow for causal inferences. Participants on the present study completed SAT, which reduce generalizability to similar populations. In addition, the low number of Latino women did not allow for an examination of gender differences within each group. Lastly, most participants were from Puerto Rican and Mexican background, which may limit generalization of findings to other Latino subcultures.

Following an ecological approach, prevention science aims to explore individual, behavioral, interpersonal, and community domains in relation to substance abuse [46, 47]. The social context (i.e., governmental policies, attitudes of native populations) in which Latinos are immersed, including social support, community and family resources may affect endorsement of cultural values and acquisition of values promoted by the host culture, which in turn inform attitudes and behaviors [48]. Given the importance of the context of reception in the acculturation process, research should investigate settings that promote *etnogenesis*, or environments that promote the blend of cultures, as potential recovery environments [49]. Community-based recovery homes, like Oxford Houses [50]. Emerge as a viable option to explore the acculturation process of individuals working on their recovery for several reasons: First, the peer-support process is likely to facilitate the learning of values and social norms needed to remain abstinent [51]. At the same time, experienced house residents provide mentorship to those who begin their recovery process, which gives more acculturated individuals the ability to share their customs and views with less acculturated individuals and vice versa. The use of guidelines, norms, and house structure not only promotes communication among house residents but also facilitates the acquisition and practice of social norms needed to navigate in the mainstream culture.

Future studies are needed to replicate studies with similar populations to ensure valid measurement of acculturation. Particularly research that test theories of acculturation using a critical lens is needed to expand current notions of acculturation and how these theories can be applied to multiple populations and settings. Given that acculturation is an evolving phenomenon, further studies on clinical samples of individuals who complete SAT are needed to test and replicate studies to inform prevention and intervention programs for specific communities, including Latinos in substance use recovery. More important, findings from substance abuse research should provide policy makers with a better understanding of the mechanisms, interpersonal dynamics, and environment conditions that impact the recovery process of Latinos with substance use disorders.

## Figures and Tables

**Table 1:** Sociodemographic characteristics of participants.

	Mean	SD
Age	36.3	10.47
Education	11.13	2.49
	Percentage	(n)
Sex		
Male	86.7	117
Female	13.3	18
Marital Status		
Married	4.6	6
Separated	17.6	23
Divorced	20.6	27
Never married	57.3	75
Country of Origin		
U. S. born (mainland)	51.9	68
Puerto Rico^1^	22.9	30
Mexico	19.8	26
Cuba	2.3	3
El Salvador	1.5	2
Guatemala	1.5	2
Generational Status		
Immigrant	44.4	60
U. S. born, both parents immigrants	28.9	39
U. S. born, 1 parent born in the U. S.	15.6	21
U. S. born, both parents born in the U. S.	11.1	15
Substance of Major Problem		
Alcohol	19.8	26
Heroin/Opiates/Analgesics	22.9	30
Cocaine	10.7	14
Cannabis/Amphetamines	9.9	13
Alcohol & one or more drugs	31.3	41
More than one, not alcohol	5.3	7
Prior Substance Abuse Treatment		
No	5.4	7
Yes	94.6	128
History of Incarceration		
No	19.1	25
Yes	80.9	110
Legal Status (on Parole/Probation)		
No	67.9	89
Yes	32.1	42

**Table 2:** Means, standard deviations, and ranges for the study variables.

Measure	1	2	3	4	5	6	7	8
1. Psychological acculturation	--							
2. Affiliation to the U. S. culture^1^	0.48**	--						
3. Affiliation to Latino culture^2^	0.53**	0.68**	--					
4. Generational status	0.34**	0.53**	−0.68**	--				
5. Alcohol use in the past 6 months	−0.15	−0.20*	0.27**	−0.22*	--			
6. Drug use in the past 6 months	−0.08	−0.05	−0.05	−0.07	0.01	--		
7. Age	−0.17*	−0.29**	−0.29**	−0.36**	0.21*	−0.24**	--	
8. Gender	0.18*	0.31**	0.40**	0.41**	−0.15	0.06	-0.24**	--
Mean	3.90	3.31	2.16	1.93	28.23	54.97	36.27	0.87
SD	1.75	0.71	0.78	1.02	45.27	54.61	10.45	--

Note.

1 Affiliation to the U. S. Culture is derived from the non-Hispanic subscale of the Bidimensional Acculturation Scale.

2 Affiliation to the Latino Culture is derived from the Hispanic subscale of the Bidimensional Acculturation Scale

** p<0.01

* p<0.05
